# Ketogenic diet ameliorates inflammation by inhibiting the NLRP3 inflammasome in osteoarthritis

**DOI:** 10.1186/s13075-022-02802-0

**Published:** 2022-05-18

**Authors:** Ganggang Kong, Jinyang Wang, Rong Li, Zhiping Huang, Le Wang

**Affiliations:** 1 Guangdong Provincial Key Laboratory of Orthopedics and Traumatology, Department of Spinal Surgery, The First Affiliated Hospital of Sun Yat-sen University, No.58, Zhong Shan Er Lu, Guangzhou, 510080 China; 2 Department of Spinal Surgery, Nanfang Hospital, Southern Medical University, Guangzhou, China; 3 Department of Pathology, Guangdong Provincial People’s Hospital, Guangdong Academy of Medical Sciences, Guangzhou, China

**Keywords:** Ketogenic diet, NLRP3 inflammasome, Osteoarthritis

## Abstract

**Background:**

The nucleotide-binding domain, leucine-rich repeat, and pyrin domain-containing protein 3 (NLRP3) inflammasome has been reported to be involved in the pathological process of osteoarthritis (OA) inflammation. Here, we investigated the ketogenic diet (KD), which has been previously demonstrated to inhibit NLRP3 inflammasome activation, to elucidate its protective mechanism against OA in rats.

**Methods:**

Anterior cruciate ligament transaction (ACLT) together with partial medial meniscectomy was used to create a rat knee joint OA model. After treatment with KD or standard diet (SD) for 8 weeks, the knee specimens were obtained for testing.

**Results:**

The KD significantly increased the content of β-hydroxybutyrate (βOHB) in rats. Compared to the SD group, the KD significantly reduced the damage caused by OA in the articular cartilage and subchondral bone. The NLRP3 inflammasome and inflammatory cytokines interleukin-1 β (IL-1β) and IL-18 were significantly increased in the SD group compared with the sham group, while their expression was significantly decreased in rats treated with the KD. In addition, MMP13 was significantly decreased in the KD group compared to that in the SD group, while COL2 was significantly increased.

**Conclusions:**

KD can protect the articular cartilage and subchondral bone in a rat OA model by inhibiting NLRP3 inflammasome activation and reducing the OA inflammatory response.

## Introduction

Osteoarthritis (OA) is a degenerative joint disorder induced by damage to the articular cartilage and subchondral bone and often leads to pain and physical disability. The major pathological feature of OA has been attributed to low-grade inflammation of the articular and peri-articular structures [1]. Pro-inflammatory cytokines, including interleukin-1 β (IL-1β), interleukin-18 (IL-18), and tumor necrosis factor α (TNF-α), have been found to accelerate the degeneration of the articular cartilage by promoting cartilage-degrading enzymes, such as aggrecanases and metalloproteinases, especially matrix metalloproteinase 13 (MMP13) expression in chondrocytes [2]. Type II collagen (COL2) and aggrecan, the main constituents of the articular extracellular matrix, demonstrate excessive degradation following disordered cartilage homeostasis [3].

The nucleotide-binding domain, leucine-rich repeat, and pyrin domain-containing protein 3 (NLRP3) inflammasome is a multiprotein platform comprising NLRP3 and the adaptor protein apoptosis-associated speck-like protein (ASC). ASC contains a caspase recruitment domain, which activates caspase-1 [4]. The activation of the NLRP3 inflammasome promotes the secretion of pro-inflammatory cytokines, including IL-1β and IL-18, and has been reported to be involved in OA [5, 6]. Increasing studies have shown that NLRP3 protein is significantly upregulated in OA cartilage. Therefore, NLRP3 inhibitors might serve as a promising strategy for OA treatment by inhibiting excessive inflammatory responses [7–9].

The ketogenic diet (KD), a high-fat, low-carbohydrate diet with adequate proteins, has been used to treat intractable epilepsy since 1921. Recent studies have shown that the KD has beneficial effects in many animal disease models, including amyotrophic lateral sclerosis, Alzheimer’s disease, traumatic brain injury, cancers, and Parkinson’s disease [10]. The ketone body β-hydroxybutyrate (βOHB) is elevated following KD treatment, and accumulating evidence suggests that βOHB is not only a carrier of energy but also acts as a signal metabolite. βOHB is a ligand for G-protein-coupled receptors that bind short-chain fatty acids, including hydroxycarboxylic acid receptor 2 and free fatty acid receptor 3, and can attenuate oxidative stress in the spinal cord and kidney by suppressing class I histone deacetylases (HDACs). Moreover, the ketone metabolite βOHB has been shown to suppress NLRP3 inflammasome-mediated inflammatory disease [11–15].

Currently, joint replacement is the only effective treatment for OA in the late stage, and no drug can significantly prevent the progression of OA. The aim of this study was to investigate whether the NLRP3 inflammasome exists in the course of OA and to further illustrate whether KD treatment improves degeneration of the articular cartilage by inhibiting NLRP3 inflammasome activation.

## Materials and methods

### Animals

All procedures in this study were approved by the Laboratory Animal Care and Use Committee of Nanfang Hospital, Southern Medical University, China. Male Sprague-Dawley rats (250–300 g) were housed (4 per cage) under standard conditions (temperature 22 °C, humidity 55%, 12/12 h cycle), with food and water provided ad libitum.

### Animal experimental design

A total of 18 rats were randomly divided into the following three experimental groups: a sham-operated (sham) group, a standard diet (SD) group, and a ketogenic diet (KD) group. The SD was provided by the Experimental Animal Center of Nanfang Hospital, Southern Medical University, and the KD with a ratio of carbohydrate and protein to the fat of 1:3 was provided by Trophic Animal Feed High-Tech Co., Ltd., China, as previously described [16]. The nutrient content of the SD and KD are listed in Table 1.

**Table 1:** Basic nutrient content of the SD and KD (per 50 g)

Component/item	SD	KD
Energy (kJ)	669.0	1402.0
Protein (g)	7.3	9.1
Fat (g)	2.0	32.6
Carbohydrates (g)	27.8	1.4
Dietary fibers (g)	2.3	3.7
Calcium (mg)	360.0	250.0
Phosphorus (mg)	300.0	150.0
Vitamin D (μg)	1.3	1.3

After being acclimated for 7 days, the rats underwent knee anterior cruciate ligament transaction (ACLT) together with partial medial meniscectomy (PMM) on the left knee joint to establish the model of OA in both the SD and KD groups and surgery without damage to joint integrity in the sham group.

The blood ketone βOHB concentration (mM) was measured by an electrochemical blood ketone monitor T-1 (Yicheng, China), and the bodyweight was measured at different times.

### Cell culture

The knee cartilage was isolated from newborn rats and cut into small pieces. The cartilage was digested with 0.25% trypsin for 30–35 min, and then 0.1% collagenase II for 5 h. Chondrocytes were collected and cultured in a medium containing 10% fetal bovine serum. Second-generation chondrocytes were used in the current study. Lipopolysaccharide (LPS)-primed chondrocytes were treated with the NLRP3 activator ATP for 60 min, following which, βOHB or MCC950 was used to treat the cells for 24 h.

### Micro-computed tomography (CT) analysis

To investigate the microstructures of the rat knee joints, after modeling for 8 weeks, the specimens were taken for a micro-CT (μCT 80, Scanco Medical, AG, Switzerland) scan. All of the joint samples were scanned at an isotropic voxel size of 15 μm, and the scanner was set at a voltage of 55 kV and a current of 145 μA. The SkyScan NRecon software was used to reconstruct the images, and the CTvox software was used to analyze the parameters of the tibial subchondral trabecular bone. The region of interest was defined as the whole subchondral bone compartment in the metaphysis of the tibia. The trabecular bone volume per tissue volume (BV/TV), trabecular number (Tb. N), trabecular thickness (Tb. Th), and trabecular separation (Tb. Sp) were calculated.

### Analysis of the general view of the knee joint

The general view of the knee joint was examined after the animals were killed. The fibrillation, erosion and ulcer formation, and osteophyte formation of the knee joint were evaluated. The macroscopic score was determined with reference to the previous literature [17].

### Histological and immunohistochemistry analyses

After decalcification with ethylene diamine tetra acetic acid for at least 4 weeks, the samples were embedded in paraffin and sectioned sagittally into 4-μm slices. Then, the slices were stained with Safranin O-fast green and hematoxylin-eosin (H&E). Three blinded reviewers evaluated the grade of degenerative changes using the Osteoarthritis Research Society International (OARSI) Scoring System. The content of glycosaminoglycan in the cartilage was evaluated by Alcian blue staining.

For immunohistochemical staining, the slices were incubated in 3% hydrogen peroxide for 30 min, before being subjected to antigen retrieval. The specimens were incubated with primary antibodies against NLRP3 (1:100, Abcam, MA, USA), ASC (1:100, Abcam, MA, USA), Caspase-1 p20 (1:100, Bioworld Technology, MN, USA), IL-1β (1:100, Bioworld Technology, MN, USA), IL-18 (1:100, Bioworld Technology, MN, USA), MMP13 (1:100, Abcam, MA, USA), and COL2 (1:100, Bioworld Technology, MN, USA) at 4 °C overnight. After washing in PBS, the slides were incubated with goat anti-rabbit HRP-conjugated secondary antibody (Boster Corporation, Wuhan, China). Finally, the reactions were developed using 3′3-diaminobenzidine. The stained sections were photographed with a light microscope (Leica DM4000 B, Leica Microsystems Inc., Germany) and were semi-quantitatively analyzed by the Image-Pro Plus software. We measured the optical density of the positively stained cells three times in randomly selected sections.

### Enzyme-linked immunosorbent assay (ELISA)

After the end of the experiment, the rats were killed by an overdose of anesthesia. The joint cavities were flushed with 1 mL 0.9% sodium chloride solution and collected as joint fluid samples. The samples were centrifuged at 4000 r/min for 5 min, and the supernatant was extracted and stored in at – 80 °C. The expression levels of IL-1β and IL-18 in the rat articular fluid were detected by ELISA (CUSABIO, Wuhan, China) in accordance with the manufacturer’s instructions.

### Western blot (WB) analysis

The knee articular cartilage tissue or rat primary chondrocytes were homogenized in ice-cold RIPA buffer with 1 mM PMSF, and protease and phosphatase inhibitors. The protein concentration of each sample was measured using the Bicinchoninic Acid Protein Assay Kit. After being separated by 10% or 12% sodium dodecyl sulfate–polyacrylamide gel electrophoresis, the protein samples were transferred to polyvinylidene fluoride membranes (Millipore, MA, USA). The blots were blocked with 5% skim milk in Tris-buffered saline with 0.1% Tween 20 for 1 h at room temperature and then incubated with primary antibodies against β-actin (1:1000, Abcam, MA, USA), Caspase-1 p20 (1:1000, Bioworld Technology, MN, USA), ASC (1:1000, Abcam, MA, USA), and NLRP3 (1:500, Abcam, MA, USA) overnight at 4 °C. The blots were then washed with TBS-T and incubated with the secondary antibody (1:5000; Bioworld Technology, MN, USA) at room temperature for 1 h. The blots were washed again with TBS-T, before visualizing with an enhanced chemiluminescence reagent (Millipore).

### Statistical analysis

All experiments were repeated at least three times. Statistical analyses were performed with the SPSS 20.0 (IBM, NY, USA) software. One-way analysis of variance (ANOVA) with the least significant difference post hoc test was used to detect differences among multiple groups. Multivariate analysis of variance (MANOVA) with repeated measures was used to detect the differences among the groups at different time points. A *P* value < 0.05 was considered statistically significant.

## Results

### Blood ketone βOHB level and body weight measurement

**Fig. 1:**
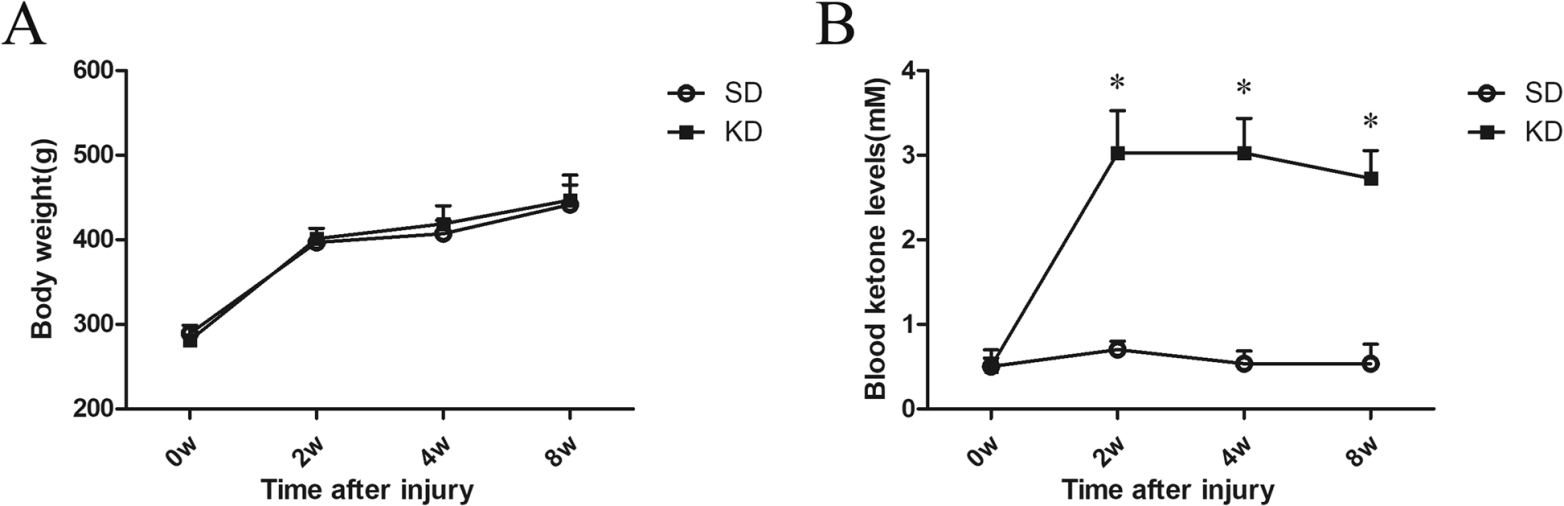
The body weight and blood βOHB levels at different times. **A** There was no significant difference in body weight between the KD and SD groups at all time points. **B** The blood βOHB levels in the KD group were significantly higher than those in the SD group. *n* = 6 per group, with a total of 12 animals used. *P < 0.05 versus the SD group

There was no significant difference in body weight between the KD and SD groups at all time points tested. The blood βOHB levels in the KD group were significantly higher than those in the SD group, and they reached an average level of 3.0 mM after feeding with KD for 2 weeks. In the SD group, the blood βOHB levels fluctuated between 0.4 and 0.8 mM (Fig. 1).

### KD improved the bone micro-architecture of the subchondral bone

**Fig. 2:**
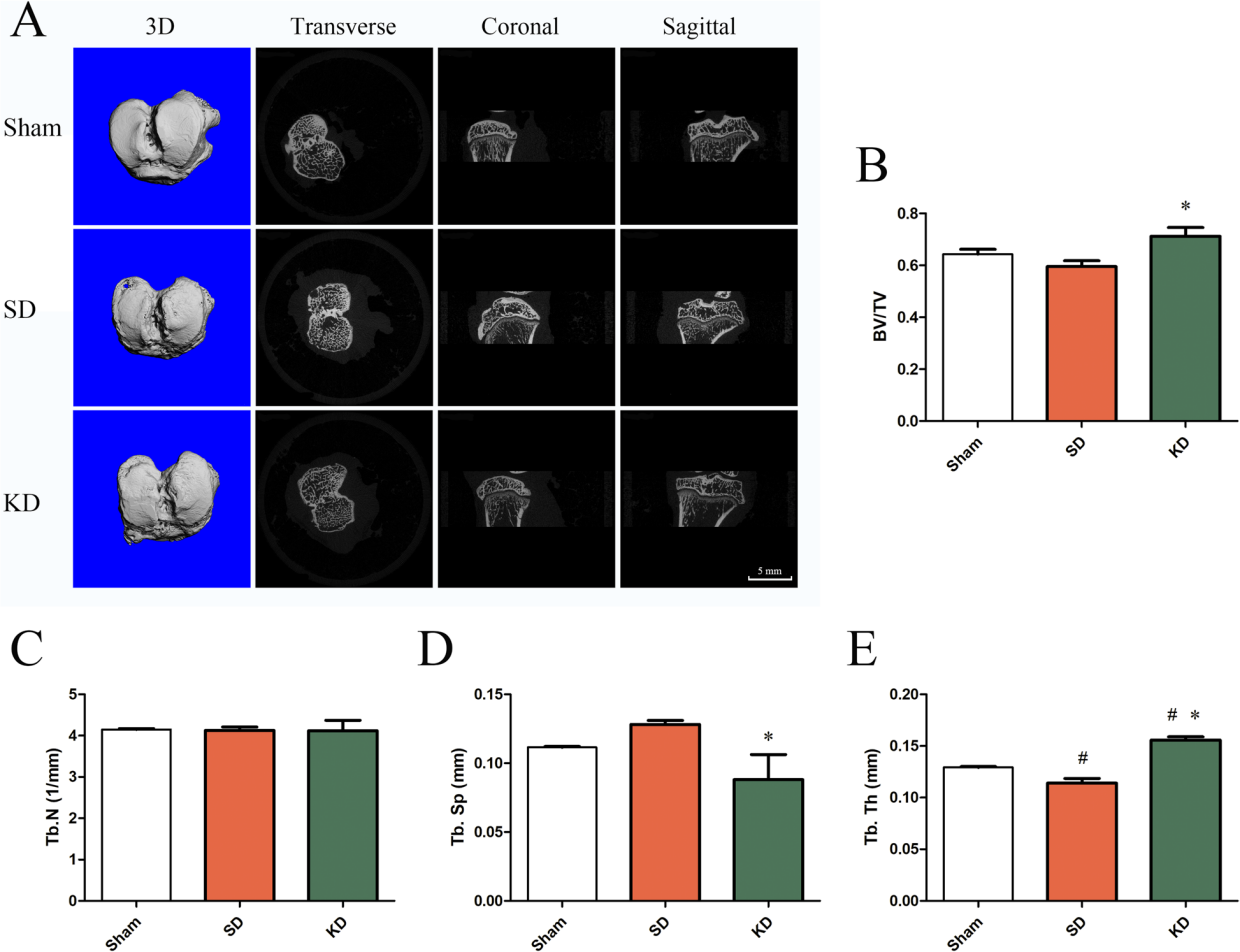
Bone micro-architecture in the subchondral bone. **A** Micro-CT images of the tibial subchondral bone at 8 weeks after surgery. Scale bar, 5 mm. **B**–**E** Quantitative analysis of the structural parameters of the subchondral bone. BV/TV, bone volume fraction; Tb. N, trabecular number; Tb. Sp, trabecular separation; Tb. Th, trabecular thickness. *n* = 6 per group, with a total of 18 animals used. ^#^*P* < 0.05 versus the sham group, *P < 0.05 versus the SD group

Compared to the SD group, the BV/TV in the KD group was significantly increased after modeling for 8 weeks. The Tb. N was not significantly different among the groups. The Tb. Sp in the KD group was significantly lower than that in the SD group, whereas the Tb. Th was significantly increased (Table 2 and Fig. 2).

**Table 2:** Changes in the bone micro-architecture parameters obtained by micro-CT

	BV/TV	Tb. N (1/mm)	Tb. Th (mm)	Tb. Sp (mm)
Sham	0.643 ± 0.026	4.150 ± 0.034	0.129 ± 0.001	0.112 ± 0.001
SD	0.595 ± 0.039	4.129 ± 0.144	0.114 ± 0.008^a^	0.128 ± 0.005
KD	0.712 ± 0.048*	4.118 ± 0.362	0.156 ± 0.004^a,b^	0.088 ± 0.026*

^a^Significant difference compared to the sham group

^b^Significant difference compared to the SD group

### Macroscopic and histopathological appearance of the knee joint

**Fig. 3:**
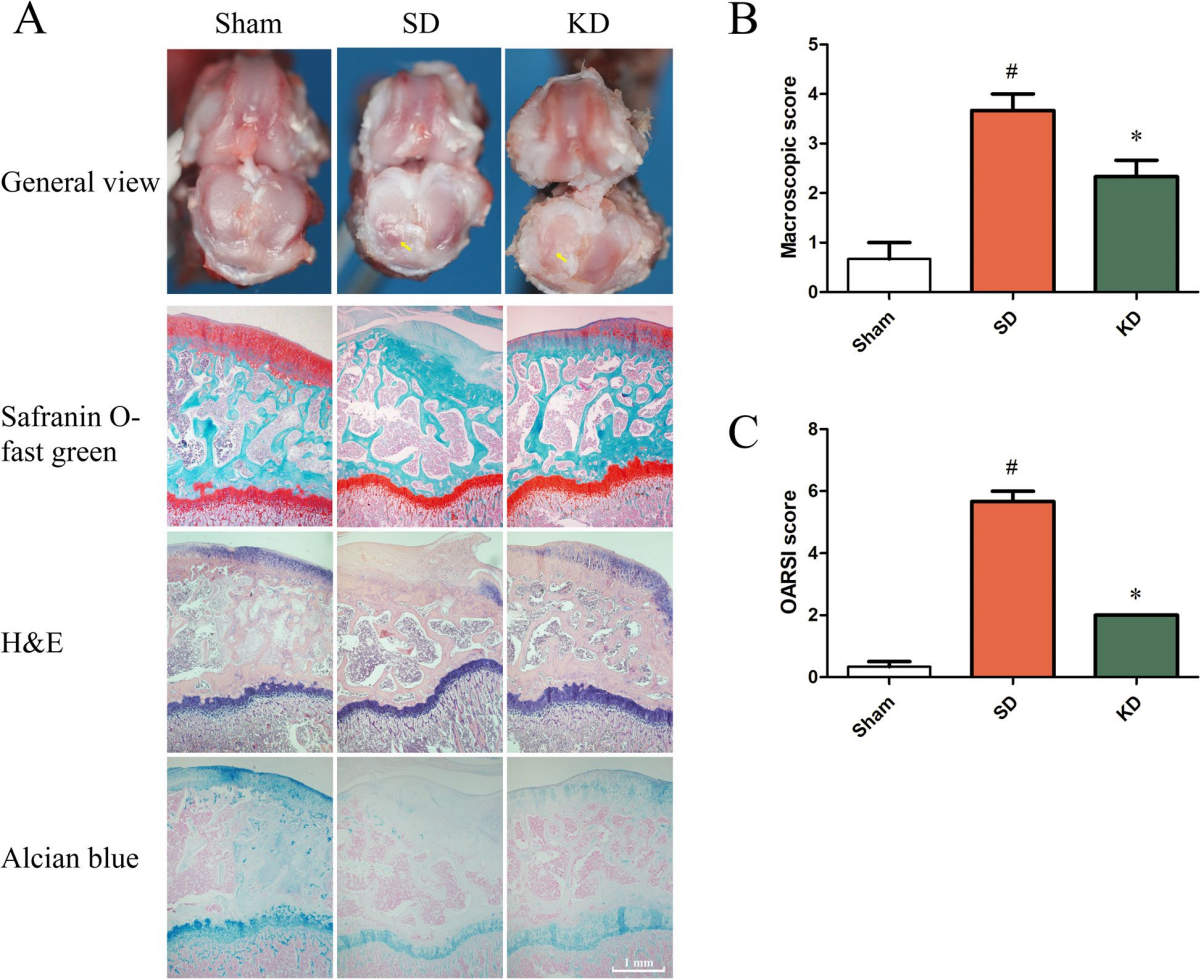
The macroscopic and histopathological appearance of the knee joints. **A** Gross observation, Safranin O-fast green, H&E, and Alcian blue staining of the knee joint at 8 weeks after surgery treatment (from top to bottom). Scale bar of histopathological images, 1 mm. **B**, **C** Macroscopic score and OARSI score of the knee joint. *n* = 3 per group, with a total of 9 animals used. ^#^*P* < 0.05 versus the sham group, *P < 0.05 versus the SD group. Ulcers are indicated using arrows

In the sham group, the cartilage surface was smooth, and no cartilage erosions or osteophytes were observed. Following surgery, the rats in the SD group showed significant articular cartilage damage, in addition to cartilage erosions and osteophytes. Notably, KD treatment significantly reduced the macroscopic score compared to that in the SD group (Fig. 3A, B).

The slices of the rat knee joint stained with Safranin O-fast green, H&E, and Alcian blue were also recorded. The results of the OARSI evaluation showed that the scores in the SD group were significantly higher than those in the sham group, while the KD significantly inhibited this change. Compared to that in the SD group, the KD increased the content of glycosaminoglycan in the knee cartilage of OA rats (Fig. 3A, C).

### KD inhibited NLRP3 inflammasome activation in OA

**Fig. 4:**
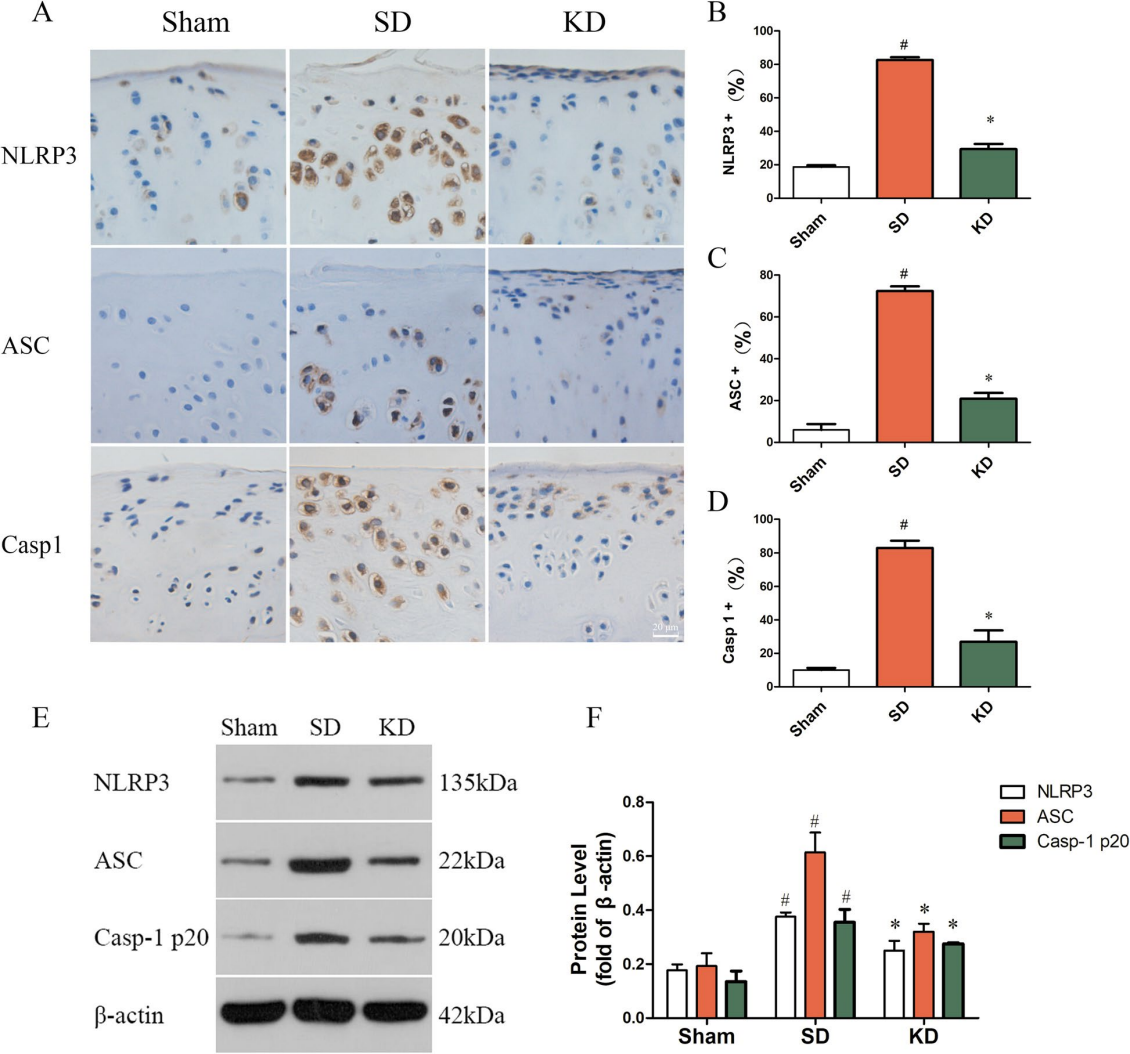
NLRP3 inflammasome activation in the knee joint. **A** The influence of KD on the activation of NLRP3 inflammasome was detected by immunohistochemistry. Scale bar, 20 μm. **B**–**D** Quantitative analysis of the expression of NLRP3 inflammasome in immunohistochemical results. **E** The expression of NLRP3 inflammasome was measured by western blot. **F** Quantitative analysis of the expression of NLRP3 inflammasome in western blot results. *n* = 3 per group, with a total of 18 animals used. ^#^*P* < 0.05 versus the sham group, *P < 0.05 versus the SD group

**Fig. 5:**
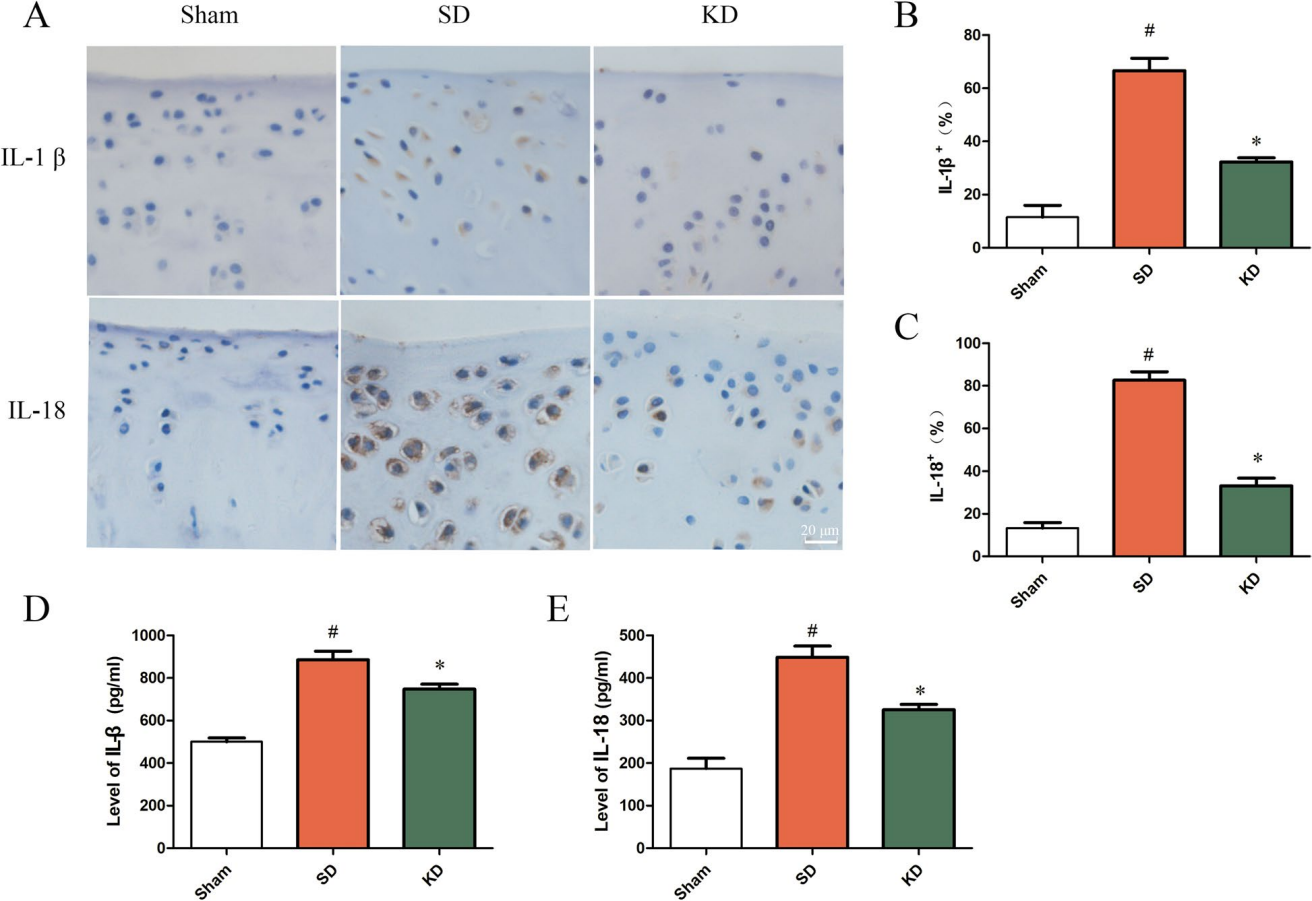
Expression of pro-inflammatory cytokines in the knee joint. **A** IL-1β and IL-18 expression levels in the articular cartilage of the knee joints, as detected by immunohistochemistry. Scale bar, 20 μm. **B**, **C** Quantitative analysis of the expression of IL-1β and IL-18 by immunohistochemistry. **D**, **E** Quantitative analysis of IL-1β and IL-18 by ELISA. *n* = 3 per group, with a total of 9 animals used. ^#^*P* < 0.05 versus the sham group, *P < 0.05 versus the SD group

NLRP3 inflammasome activation was detected by immunohistochemistry and WB (Fig. 5A). The findings showed that OA significantly increased the protein expression of NLRP3, ASC, and caspase-1 p20. However, KD intervention significantly inhibited the high expression of these proteins compared to that observed following the SD (Fig. 4).

### KD reduced the levels of pro-inflammatory cytokines in OA

The expression levels of the key inflammatory markers IL-1β and IL-18 were analyzed by immunohistochemistry and ELISA. Immunohistochemical results showed that the KD significantly inhibited the increase in the proportion of IL-1 and IL-18-positive cells induced by OA. The results of ELISA were similar, indicating that OA increased the secretion of IL-1β and IL-18 in the articular fluid and the KD significantly inhibited the secretion of IL-1β and IL-18 compared to that in the SD group (Fig. 5).

### KD inhibited degeneration of the articular cartilage in OA

**Fig. 6:**
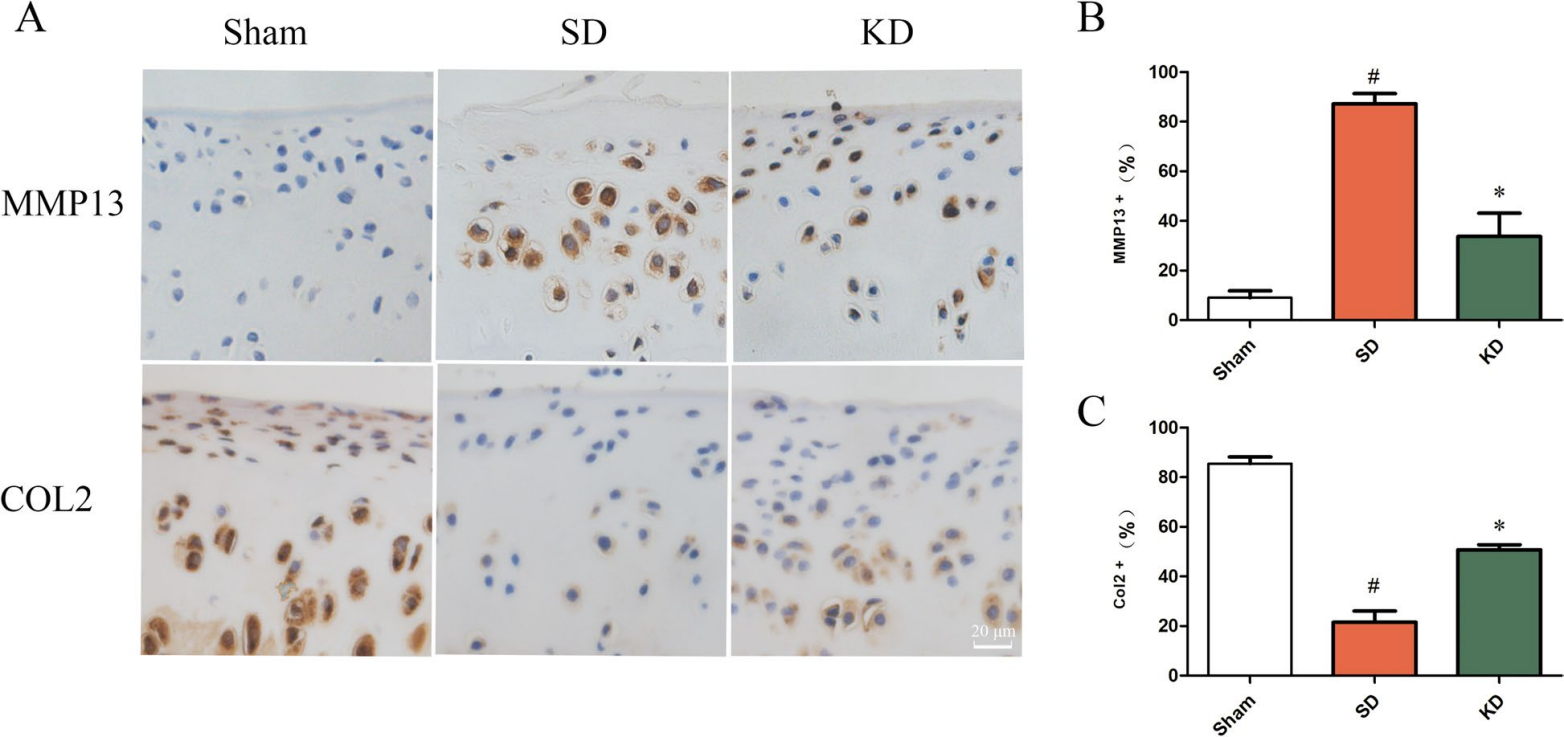
Evaluation of cartilage degeneration in the knee joint. **A** Expression of MMP13 and COL2 was detected using immunohistochemistry. Scale bar, 20 μm. **B**, **C** Quantitative analysis of the expression of MMP13 and COL2 in immunohistochemical results. *n* = 3 per group, with a total of 9 animals used. ^#^*P* < 0.05 versus the sham group, *P < 0.05 versus the SD group

The proportion of MMP13-positive cells in the KD group was significantly lower than that in the SD group, while the proportion of COL2-positive cells was significantly higher (Fig. 6).

βOHB inhibited the expression of inflammatory cytokines and MMP13 in chondrocytes through NLRP3.

**Fig. 7:**
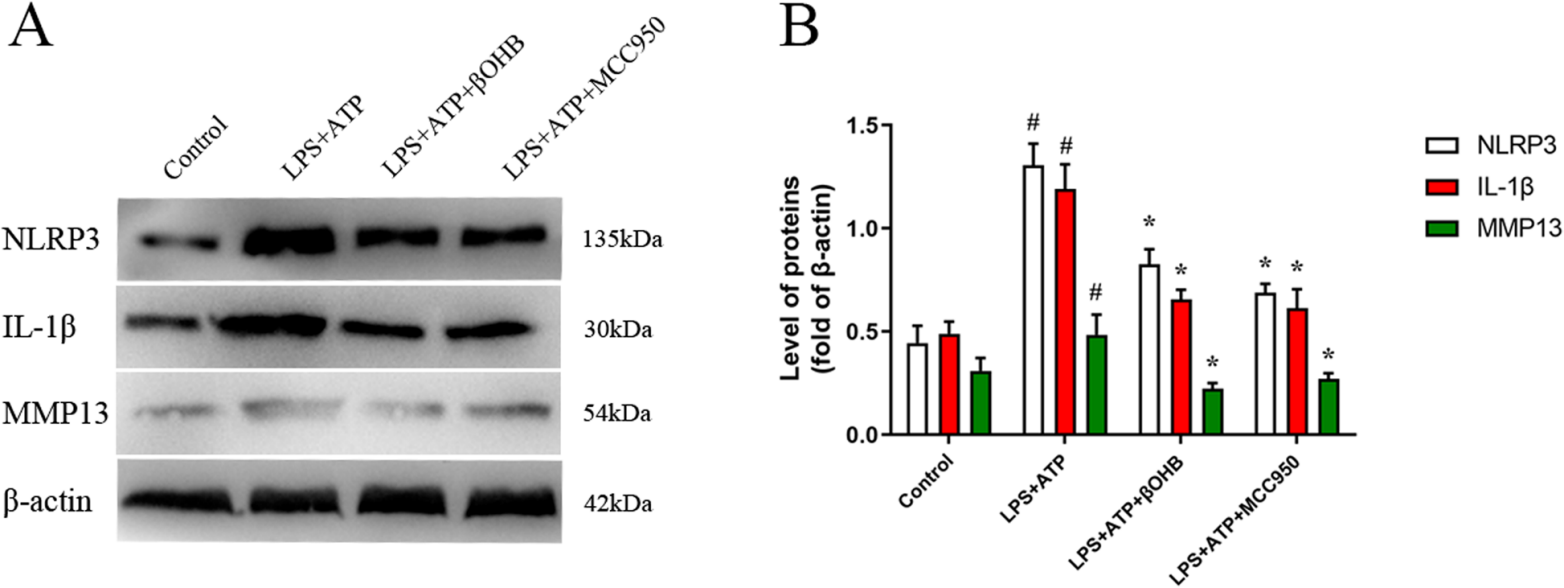
βOHB inhibited inflammatory cytokine and MMP13 expression in chondrocytes through NLRP3. **A** NLRP3, IL-1β, and MM13 were detected in the cell lysates by western blot. **B** Quantification of NLRP3, IL-1β, and MM13 in western blot results. ^#^*P* < 0.05 versus the control group, *P < 0.05 versus the LPS+ATP group

LPS-primed chondrocytes were treated with the NLRP3 activator ATP. βOHB or NLRP3 inhibitor MCC950 treatment downregulated NLRP3 and IL-1β expression, thereby reducing the expression of MMP13 (Fig. 7).

## Discussion

In this study, conventional ACLT+ PMM surgery was used to establish a rat OA model, which has the advantages of good repeatability, short modeling time, a high success rate of modeling, and the ability to simulate human OA to a large extent. Eight weeks after the operation, the knee joints of rats in the SD group showed cartilage erosions and osteophytes, while the immunohistochemical results showed significant destruction of the articular cartilage, which confirmed the successful establishment of the OA model in rats.

OA is a common age-related joint disease, with unclear pathogenesis. However, increasing evidence supports the claim that inflammation is a pathological factor of the OA process. Indeed, the levels of pro-inflammatory cytokines are significantly increased in synovial fluids from patients with OA. Among them, IL-1β is considered to be the most important inflammatory factor in the pathogenesis of OA, which can further promote the release of other inflammatory substances, aggravate the inflammatory response, and eventually accelerate the destruction of the articular cartilage [18]. The effect of IL-1β on the degradation of the articular cartilage is attributed to the promotion of cartilage matrix degradation. Upregulation of IL-1β leads to high expression of MMPs, especially MMP13, which leads to irreversible destruction of the cartilage matrix [19]. Our results confirmed that OA increased the secretion of the pro-inflammatory cytokines IL-1β and IL-18 in the articular fluid, increased the expression of MMP13 in the articular cartilage, and decreased the level of COL2, which is consistent with the findings of previous studies.

Anti-inflammatory therapy is considered to be an effective treatment for OA. Silent chondrocytes can be activated by uncontrolled inflammation and secrete proinflammatory cytokines, which also express multiple inflammatory cytokine receptors. Excessive activation of inflammation increases the expression of collagenases and aggrecanases and eventually leads to degradation of the extracellular matrix of the cartilage [20–22].

While ketone bodies have long been considered a source of circulating energy during fasting, their signaling function has only recently been recognized [13]. Recent studies have shown that ketone bodies play an anti-inflammatory role in numerous diseases [23]. A study by Rahman et al. showed that KD or βOHB induced a neuroprotective phenotype in monocytes and/or macrophages through HCA2 [24]. Zhang et al. demonstrated that ketogenesis can inhibit inflammatory macrophage activation, indicating its potential therapeutic effect in acute pancreatitis [25]. In addition, the ketone body βOHB downregulates LPS-induced iNOS, COX-2, TNF-α, IL-1β, and IL-6 expression by GPR109A in murine microglial BV-2 cells [26]. We previously used the KD to treat spinal cord injury rats, suggesting that βOHB can promote the recovery of spinal cord nerve function by inhibiting the inflammatory response [11]. The implementation of a KD can effectively increase the level of the ketone body, but whether it can protect articular cartilage by inhibiting inflammation in OA rats remains unclear. The current study showed that the KD significantly reduced the secretion of pro-inflammatory factors, reduced the degradation of COL2 by inhibiting the activity of MMP13, and improved trabecular bone alterations in the metaphysis, with increases in BV/TV and Tb. Th and decreases in Tb. Sp. Finally, KD treatment significantly reduced the knee cartilage injury score of OA rats. These results confirmed the protective effect of KD on articular cartilage in OA rats, although further study is needed to elucidate its mechanism. A study by Wu X. suggested that KD compromises both the cancellous and cortical bone architecture of long bones. However, their study was significantly different from the current study in that their results were obtained by feeding mice with a KD for 12 weeks. The use of a long-term KD in mice may have contributed to the decline in BV/TV in their study, and more research is needed to confirm this hypothesis [27].

Youm et al. found that the ketone body βOHB inhibited the activation of the NLRP3 inflammasome in murine bone marrow macrophages by inhibiting intracellular K^+^ outflow and ASC oligomerization [28]. Furthermore, βOHB improved NLRP3 inflammasome-mediated inflammatory diseases, such as Muckle-Wells syndrome, spinal cord injury, and gout flares, and reduced caspase-1 activation and IL-1β secretion. Wu et al. used βOHB to treat osteolysis induced by CoCrMo Alloy particles and found that βOHB could exert anti-inflammatory effects through the NLRP3 inflammasome and regulate the function of osteoclasts, thus relieving osteolysis [29]. It was concluded that KD inhibited the inflammatory response of OA through the NLRP3 inflammasome, thus protecting the articular cartilage.

The inflammasome is a protein complex found in the cytoplasm, and it is involved in the regulation of the inflammatory response. The NLRP3 inflammasome is the most widely studied NLR family member, it can be activated not only by pathogens, such as bacteria and viruses, but also by various risk signals and metabolites in the body, including ATP, urate, and amyloid beta-protein [30]. Studies have shown that the NLRP3 inflammasome plays an important role in the inflammatory pathogenesis of OA and may represent a new therapeutic target [5, 31]. The NLRP3 inflammasome is activated in OA and promotes the maturation and secretion of IL-1β and IL-18 [32]. Increasing studies have shown that inhibition of NLRP3 inflammasome activation in OA articular cartilage can reduce the inflammatory response and thus reduce articular cartilage injury [7–9]. Ni et al. showed that the NLRP3 inhibitor MCC950 can alleviate articular cartilage degradation in a mouse OA model [7]. Bai et al. demonstrated that intraarticular injection of baicalein inhibited cartilage catabolism through the NLRP3 inflammasome [8]. Similarly, our study showed that the NLRP3 inflammasome was highly expressed in OA of the knee joint of rats and that KD treatment significantly inhibited the activation of the NLRP3 inflammasome in the articular cartilage of OA rats. Similar to the NLRP3 inhibitor MCC950, the ketone βOHB also inhibited ATP-induced NLRP3 activation, resulting in decreased inflammatory cytokine IL-1β and the cartilage matrix-degrading enzyme MMP13 expression in primary chondrocytes. Our findings highlight that KD protects OA articular cartilage by inhibiting inflammation through NLRP3.

## Conclusions

Our results showed that KD treatment could reduce knee inflammation by inhibiting the activation of the NLRP3 inflammasome, thereby reducing articular cartilage and subchondral bone damage in OA rats. This study may provide a new strategy for the treatment of OA.

## Data Availability

Not applicable.

## Abbreviations

OA: Osteoarthritis
IL-1β: Interleukin-1 β
IL-18: Interleukin-18
TNF-α: Tumor necrosis factor α
MMP13: Matrix metalloproteinase 13
COL2: Type II collagen
NLRP3: The nucleotide-binding domain, leucine-rich repeat, and pyrin domain-containing protein 3
ASC: Apoptosis-associated speck-like protein
KD: Ketogenic diet
βOHB: β-Hydroxybutyratehistone deacetylases (HDACs)
SD: Standard diet
ACLT: Anterior cruciate ligament transaction
PMM: Partial medial meniscectomy
CT: Computed tomography
BV/TV: Trabecular bone volume per tissue volume
Tb. N: Trabecular number
Tb. Th: Trabecular thickness
Tb. Sp: Trabecular separation
OARSI: Osteoarthritis Research Society International
ELISA: Enzyme-linked immunosorbent assay
WB: Western blot
ANOVA: One-way analysis of variance
MANOVA: Multivariate analysis of variance
